# Infantile Scabies in the Democratic Republic of Congo: Observations From the First Case Report

**DOI:** 10.1002/ccr3.70220

**Published:** 2025-02-17

**Authors:** Nambininiavo Marianne Ranorohasimanana, Arezki Izri, Sophie Brun, Philippe Parola, Mohammad Akhoundi

**Affiliations:** ^1^ Parasitology‐Mycology Department, Avicenne Hospital, AP‐HP Sorbonne Paris Nord University Bobigny France; ^2^ Aix‐Marseille Univ Marseille France; ^3^ Unité Des Virus Émergents (UVE: Aix‐Marseille Univ, Università di Corsica, IRD 190, Inserm 1207, IRBA) Marseille France

**Keywords:** Democratic Republic of Congo, irritability, rashes, *Sarcoptes*, scabies

## Abstract

Scabies is common in the Democratic Republic of Congo, often underestimated, including in infants. In this report, a 4‐month‐old's irritability, nocturnal itching, insomnia, and skin rashes raised suspicion, confirmed by parasitological examination. The father's lack of infestation despite sharing the bed with the infant and his mother poses a question.

## 1 Introduction

Scabies is a parasitic disease caused by the mite *Sarcoptes scabiei* variety hominis. It occurs worldwide, affecting approximately 200 million individuals per year, with a higher prevalence *in tropical and subtropical areas*. According to the World Health Organization (WHO), scabies is considered a neglected tropical disease with a global prevalence ranging from 0.2% to 71.4% [1]. Recently, an overall prevalence of scabies ranging from 0.18% to 76.9% has been reported in children [2]. Scabies is often spread through a relatively prolonged direct skin‐to‐skin contact with an infected person. Although sexual contact is reported as the most common mode of transmission, spread through contact with infested personal items (e.g., towels, clothes, bed linens) is also reported. The risk of spread increases in crowded living conditions such as those found in childcare facilities, clustered homes, retirement homes, refugee camps, soldier camps, and prisons.

The common symptoms of the disease include pruritus, pimple‐like rash, papules, or pustules, sometimes with tiny burrows in the skin. In infants and young children, scabies manifests as vesicles, pustules, nodules, and pruritus, often with a generalized eruption involving the head and face, diaper region, scalp, palms, and soles [3]. Pruritus can be very severe, and infants may be irritable [3]. Scratching may cause skin breakdown and additional bacterial infection of the skin or post‐infectious complications.

Diagnosis of scabies in young children can be challenging because adult scabies and infantile scabies present differently in their morphologies, distribution, and symptoms. It is particularly difficult to distinguish burrows among the vesicular and eczematous lesions of scabies in children, notably infants [4]. In infants < 2 years old, the diagnosis can be even more difficult due to its clinical presentation resembling many other dermatoses, sometimes leading to misdiagnosis.

In this report, we present a case of an infant infested with *Sarcoptes* mites in the Democratic Republic of Congo (DRC).

## 2 Case History/Examination

A 4‐month‐old boy was referred by his mother in December 2023 to Ngamanzo Hospital, in the northern suburb of Kinshasa (capital of DRC), due to a cutaneous eruption. He was the only infant in the family who lived with his parents in the village of Ngamanzo, approximately 55 km from the capital, where according to local health authorities, there were many cases of scabies infestation. The family used to sleep altogether on a sponge mattress placed on the floor. Moreover, the infant was showering twice a day and wearing socks. Additionally, there was a notion of itching and bedbug infestation at home. The infant was afebrile with a good general condition.

**FIGURE 1:**
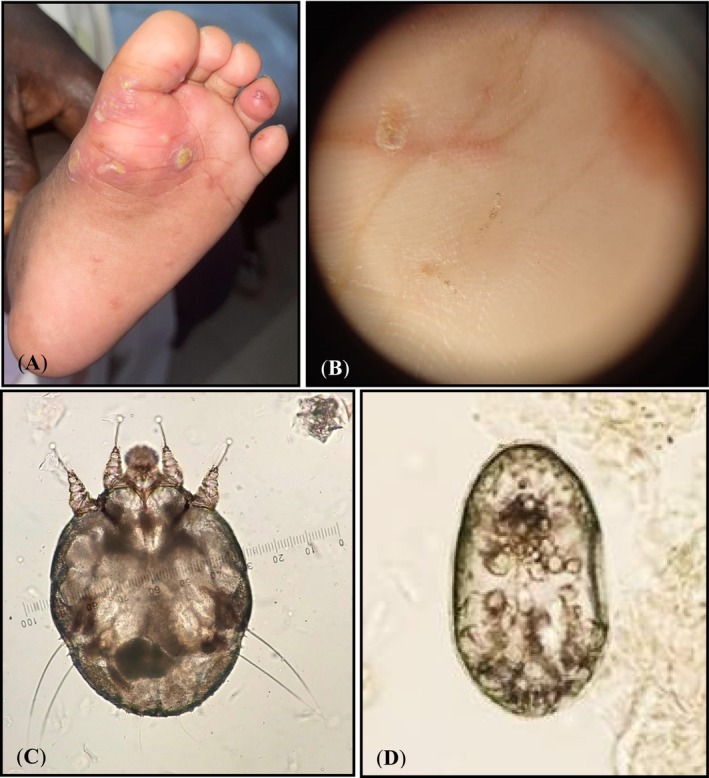
Scabies infestation on the soles of the feet in a 4‐month‐old male patient (A); scabies burrow with small irregular tracks (B); female adult of *Sarcoptes scabiei* (C); and embryonated egg (D) isolated from the infant and his mother.

Initial clinical examination of the infant and interrogation of the parents revealed irritability, insomnia, pustule‐like skin rashes on the soles of the feet and palms of the hands, associated with multiple scaly lesions on the wrist and interdigital spaces that had been exhibited for a month (Figure 1A,B).

His mother was clinically examined as well, presenting erythemato‐papular eruptions all over the body and scaly lesions on the wrists and interdigital spaces with recurrent nocturnal pruritus. Conversely, his father showed no clinical signs of scabies infestation.

Parasitological examination using a dermatoscope revealed the scabies burrows on the palm of the right hand. The female *S. scabiei* mites and eggs were isolated from the infant and his mother after microscopic examination under 100× magnification of scale scraping (Figure 1C,D).

### 2.1 Differential Diagnosis

Symptoms such as irritability and insomnia, along with severe pruritic and polymorphic skin lesions, allow for differentiating infantile scabies.

### 2.2 Conclusion and Results

Treatment with the topical application of ascabiol (benzyl benzoate 10%) on Day (D) 0 and D8 for the infant and with ivermectin (200 μg/kg) on D0 and D10 for the parents prevented recurrence of infestation and resulted in a favorable outcome 2 weeks later.

## 3 Discussion

The DRC is a tropical country where scabies is widespread [5]. Despite this medical significance, very little is known about scabies in this country, and various clinical and epidemiological aspects of the disease remain largely unknown. Based on the literature, only two surveys have been carried out in this country. In the first survey, performed in Kinsenso, a semi‐urban county of Kisenso in Kinshasa, 3.95% of the Congolese schoolchildren population (*N* = 2024) was infested with scabies. Boys (56% vs. 44% for girls) and interdigital spaces (93.8%) were found to be the most affected individuals and body parts, respectively [6]. In another survey carried out on the patients referred from 2 January 2008 to 31 August 2017 to the University‐Hospital of Kinshasa, 41.9% of individuals examined were infested with scabies [7]. In this report, we officially document for the first time a case of infantile scabies in a 4‐month‐old boy with infestation on the soles of the feet and palms of the hands (Figure 1A,B). In addition, his mother was also infested with scabies, exhibiting lesions all over the body and scaly lesions on the wrists and interdigital spaces. Regarding the appearance time of clinical symptoms in the infant and his mother, and according to the parents' statement, it is hypothesized that the infant was likely infested by his mother while sleeping with her or feeding with milk. On the other hand, given the prolonged period of infestation in the infant and considering that all family members, including the father, shared the same bed, the absence of infestation in the father raises a question.

Diagnosing infantile scabies can indeed be challenging due to variations in the morphology, distribution of skin lesions, and clinical appearance in infants compared to adults. Infants often present with inflammatory burrows characterized by red, edematous, crusted, oval to elongated, serpiginous, or J‐shaped papulovesicles and nodules, bullous and vesicular lesions on soles accompanied by nocturnal pruritus, insomnia, and irritability, and exhibited early in the course of infestation [8]. In infants < 2 years of age, scabies often presents with axillary or inguinal nodules and with papulovesicles and pustules on the dorsum of the foot, palms, scalp, and face [9]. There is often an absence of excoriations because infants have not fully developed the ability to scratch. In the case of our patient, symptoms such as irritability, nocturnal pruritus, insomnia, and skin rashes on the soles of the feet and palms of the hands raised suspicion of scabies infestation, which was confirmed further by parasitological examination. Therefore, clinicians should be mindful of the differential diagnosis such as irritability and insomnia in an infant presenting with severe pruritic and polymorphic skin lesions.

The treatment of scabies has evolved with the development of new anti‐ectoparasitic strategies. Medications such as benzyl benzoate, ivermectin, or permethrin have been demonstrated to be effective in treating scabies [10]. Benzyl benzoate is a well‐known antiparasitic agent used in many countries to treat scabies and lice. It is one of the recommended treatments in the European guidelines for the management of scabies and is recommended as the “first‐line intervention” for the cost‐effective treatment of the disease [11]. This acaricide is believed to be absorbed by *Sarcoptes* mites, destroying them by acting on their nervous system. Benzyl benzoate 10% is approved for topical application from the age of 1 month and 25% in children 12 years or older [12]. Furthermore, ivermectin is another effective antiparasitic agent that acts against various parasitic diseases, including strongyloidiasis, scabies, lice infestations, gnathostomiasis, and myiasis. In the case of our patient, the topical application of benzyl benzoate was demonstrated to be effective in the treatment of infantile scabies, without any notion of irritation, and resulted in a favorable outcome 2 weeks post‐treatment. Likewise, ivermectin was successful in treating the scabies of the parent.

## Author Contributions

**Nambininiavo Marianne Ranorohasimanana:** conceptualization, investigation, methodology, writing – review and editing. **Arezki Izri:** investigation, methodology, writing – review and editing. **Sophie Brun:** investigation, methodology, writing – review and editing. **Philippe Parola:** investigation, methodology, writing – review and editing. **Mohammad Akhoundi:** conceptualization, data curation, investigation, methodology, writing – original draft, writing – review and editing.

## Ethics Statement

Ethical approval for this study was granted through protocol number 95/99/AVC/ESA by the Avicenne Hospital Research Ethics Committee.

## Consent

A written consent was provided and signed by the parents, including the authorization for publishing the clinical information.

## Conflicts of Interest

The authors declare no conflicts of interest.

## Data Availability

All data and related information underlying reported findings have been presented in the article.
